# Environmental Toxicology and Metabolism

**DOI:** 10.3390/metabo14100530

**Published:** 2024-09-30

**Authors:** Yu Sun

**Affiliations:** 1 State Key Laboratory of Swine and Poultry Breeding Industry, South China Agricultural University, Guangzhou 510642, China; sunyu@scau.edu.cn; 2 Guangdong Provincial Key Laboratory for the Development Biology and Environmental Adaptation of Agricultural Organisms, College of Life Sciences, South China Agricultural University, Guangzhou 510642, China

The modern world is witnessing an unprecedented rise in environmental exposures to hazardous substances such as pesticides, heavy metals, and synthetic chemicals. Rapid industrialization, intensive agricultural practices, and globalization have led to the widespread distribution of these toxicants in the environment, posing significant risks to human health and ecosystems [1]. Pesticides, essential for modern agriculture, often contaminate air, soil, and water, leading to chronic exposure among populations [2]. Heavy metals like cadmium, lead, and mercury, which are released from industrial activities and improper waste disposal, accumulate in the environment and can bioaccumulate in food chains [3]. Synthetic chemicals, including preservatives, plasticizers, and flame retardants, are ubiquitous in consumer products, resulting in continuous low-level exposure [4].

Understanding the metabolic and health impacts of these environmental exposures is crucial for developing effective interventions and policies to safeguard public health. Chronic exposure to pesticides has been linked to endocrine disruption, neurotoxicity, and metabolic disorders such as obesity and diabetes [5,6]. For example, bisphenol A (BPA), a widely used industrial chemical found in plastics, has been linked to insulin resistance and obesity by interfering with pancreatic β-cell function and adipogenesis [7]. Organophosphate pesticides can inhibit acetylcholinesterase, leading to neurodevelopmental deficits and metabolic alterations [8]. Heavy metals are known to interfere with essential metabolic enzymes, generate oxidative stress, and cause hepatotoxicity and nephrotoxicity [9]. Cadmium and lead have been associated with hypertension, renal dysfunction, and disruptions in lipid and glucose metabolism [9]. Cadmium exposure, in particular, has been associated with disruptions in gut microbiota composition, contributing to metabolic disorders [10]. A study by Tinkov et al. (2017) highlighted the role of cadmium in obesity and metabolic syndrome, emphasizing the need for monitoring environmental exposure levels [11].

Moreover, the human and environmental microbiota play a pivotal role in mediating the effects of environmental toxicants. Alterations in microbial communities, whether in the gut, oral cavity, or environmental settings, are often linked to diseases such as metabolic dysfunction-associated steatotic liver disease (MASLD), allergies, asthma, obesity, diabetes, and gastrointestinal disorders [12,13,14,15,16,17]. Environmental exposures can disrupt microbiota diversity, leading to dysbiosis and influencing immune responses, metabolism, and disease susceptibility [18,19]. For example, exposure to polychlorinated biphenyls (PCBs) altered the gut microbiome composition in mice, contributing to metabolic disorders [20]. Exposure to heavy metals and pesticides can alter gut microbiota composition, affecting short-chain fatty acid production and intestinal barrier function [21]. Similarly, a study in China, which was conducted as two cross-sectional analyses at different time points, demonstrated that changes in outdoor air pollutants influenced the indoor microbiome, which mediated health effects such as asthma, rhinitis, and eczema in children [19].

This Special Issue of *Metabolites* brings together cutting-edge research that delves into the intricate relationships between environmental toxicants, microbiota, metabolic processes, and therapeutic strategies. By exploring these complex interactions, the included studies contribute valuable insights that can inform public health policies and interventions aimed at mitigating the adverse effects of environmental exposures.

Environmental toxins such as pesticides, heavy metals, and synthetic chemicals pose significant risks to both human health and ecosystems. In this Special Issue, several studies explore the metabolic consequences of such exposures. Groswald et al. investigate the hepatotoxic effects of the insecticide methomyl in mice, revealing disruptions in xenobiotic metabolism and increased hepatic steatotic liver disease (MASLD). Their study demonstrates that methomyl exposure leads to metabolic disturbances beyond its known cholinesterase inhibition, highlighting the insecticide’s broader impact on liver health and metabolism (contribution 1). Yang et al. examine how ecologically relevant concentrations of cadmium alter gut microbiota and short-chain fatty acids in zebrafish, shedding light on potential mechanisms of neurodevelopmental toxicity. This study links low-level cadmium exposure to specific changes in gut microbiota and signaling pathways, suggesting a gut–brain axis involvement in cadmium-induced neurotoxicity (contribution 2). Nolasco et al. provide insights into the metabolic alterations in workers occupationally exposed to pesticides, identifying potential biomarkers that could aid in early detection and intervention. Utilizing a metabolomics approach, their research uncovers alterations in lipid and amino acid metabolism, offering valuable biomarkers for monitoring pesticide exposure and its health effects in occupational settings (contribution 3). Jo et al. assess the pharmacokinetics of benzisothiazolinone (BIT) in rats, contributing valuable data for risk assessments related to human exposure. Their comprehensive study on BIT’s absorption, distribution, metabolism, and excretion fills a critical gap in understanding the systemic exposure and potential health risks of this widely used preservative (contribution 4). Fu et al. highlight the presence of microbial virulence factors and synthetic chemicals in aircraft cabins, emphasizing the need for improved environmental controls in air travel to protect passenger health. Their multi-omic analyses uncover the abundance of pathogenic microbes and harmful chemicals in the airline environment, underscoring the importance of monitoring and mitigating these exposures to safeguard public health during air travel (contribution 5).

The human and environment microbiota play a pivotal role in maintaining health, with alterations in microbial communities often being linked to various diseases. Li et al. demonstrate that Astragaloside IV not only protects against noise-induced hearing loss in mice, but also modulates intestinal flora and reduces inflammatory cytokines. They illustrate how the modulation of gut microbiota by Astragaloside IV can attenuate inflammation and prevent auditory damage, proposing a novel therapeutic strategy for noise-induced hearing loss through microbiota regulation (contribution 6). Zhang et al. explore how the indoor microbiome and metabolites influence children’s nasal and oral microbiota, identifying factors that could promote respiratory health or increase disease risk. Their pilot multi-omic analysis reveals the transfer of specific microbes and metabolites from indoor environments to children, highlighting the impact of environmental microbiomes on pediatric respiratory health and the potential for interventions targeting indoor environments (contribution 7). AlMalki et al. study the metabolic alterations in breast cancer cells exposed to *E. coli* secretome, providing a model for understanding microbiota–tumor interactions. Their research demonstrates how bacterial metabolites can induce significant metabolic changes in cancer cells, offering insights into the role of microbiota in cancer metabolism and suggesting new avenues for therapeutic development (contribution 8). The study by Yang et al. also contributes to this theme by showing how cadmium exposure affects gut microbiota in zebrafish, reinforcing the concept of environmental toxicants influencing health through microbiota alterations (contribution 2). Fu et al. reveal that aircraft cabins harbor microbial virulence factors and antimicrobial resistance genes, highlighting potential health risks associated with microbial exposure during flights. Their findings stress the importance of understanding environmental microbiomes in public transportation and their implications in the spread of infectious diseases (contribution 5).

In the quest for effective interventions against environmental stressors, identifying protective agents is of paramount importance. Li et al. showcase the therapeutic potential of Astragaloside IV in preventing hearing loss and reducing inflammation through gut microbiota modulation. Their study is particularly significant for the proposal that Astragaloside IV, as a natural compound, can modulate the gut–immune axis, offering a promising approach for preventing and treating noise-induced auditory damage (contribution 6). Wu et al. delve into the interactions between azole antifungal drugs and the human enzyme AKR7A2, revealing how certain drugs can enhance enzyme activity and potentially influence drug metabolism. Their work uniquely identifies the specific binding and activation effects of azoles on AKR7A2, contributing to our understanding of drug–enzyme interactions and informing safer use of azole antifungals in clinical setting (contribution 9). These studies underscore the potential of leveraging biochemical interactions for therapeutic benefit and highlight the need for continued research into metabolic modulation strategies.

The diverse studies featured in this Special Issue illuminate the complex interplay between environmental factors, metabolic processes, and health outcomes. By integrating insights from toxicology, microbiology, pharmacology, and biochemistry, these papers advance our understanding of how environmental exposures influence metabolism and how we might mitigate their adverse effects. Notably, several studies highlight the crucial role of microbiota modulation and biochemical interactions in developing effective interventions. As we move forward, it is imperative to consider factors such as sex and gender differences in the outcomes of environmental pollution on human health, as these can significantly influence susceptibility and response to toxicants. Additionally, embracing interdisciplinary research and multi-omic approaches will be essential in unraveling the complex mechanisms underlying environmental toxicant effects. We hope this collection inspires further interdisciplinary research to address the pressing challenges posed by environmental toxicants in our rapidly changing world, ultimately informing public health policies and interventions that protect and promote human health.

## Data Availability

Not applicable.
